# Clumps of Mesenchymal Stem Cells/Extracellular Matrix Complexes Generated with Xeno-Free Chondro-Inductive Medium Induce Bone Regeneration via Endochondral Ossification

**DOI:** 10.3390/biomedicines9101408

**Published:** 2021-10-07

**Authors:** Susumu Horikoshi, Mikihito Kajiya, Souta Motoike, Mai Yoshino, Shin Morimoto, Hiroki Yoshii, Tomoya Ogawa, Hisakatsu Sone, Tomoyuki Iwata, Kazuhisa Ouhara, Shinji Matsuda, Noriyoshi Mizuno, Hidemi Kurihara

**Affiliations:** Department of Periodontal Medicine, Graduate School of Biomedical and Health Sciences, Hiroshima University, 1-2-3 Kasumi, Minami-Ku, Hiroshima 734-8553, Japan; horiko@hiroshima-u.ac.jp (S.H.); soutamotoike@gmail.com (S.M.); myoshino@hiroshima-u.ac.jp (M.Y.); m18548@hiroshima-u.ac.jp (S.M.); hyoshii@hiroshima-u.ac.jp (H.Y.); tomoya@hiroshima-u.ac.jp (T.O.); sone@hiroshima-u.ac.jp (H.S.); iwatat@hiroshima-u.ac.jp (T.I.); kouhara@hiroshima-u.ac.jp (K.O.); matsudas@hiroshima-u.ac.jp (S.M.); mizuno@hiroshima-u.ac.jp (N.M.); hkuri@hiroshima-u.ac.jp (H.K.)

**Keywords:** chondrogenic induction, bone regeneration, endochondral ossification, scaffold-free, C-MSCs

## Abstract

Three-dimensional clumps of mesenchymal stem cells (MSCs)/extracellular matrix (ECM) complexes (C-MSCs) can be transplanted into tissue defect site with no artificial scaffold. Importantly, most bone formation in the developing process or fracture healing proceeds via endochondral ossification. Accordingly, this present study investigated whether C-MSCs generated with chondro-inductive medium (CIM) can induce successful bone regeneration and assessed its healing process. Human bone marrow-derived MSCs were cultured with xeno-free/serum-free (XF) growth medium. To obtain C-MSCs, confluent cells that had formed on the cellular sheet were scratched using a micropipette tip and then torn off. The sheet was rolled to make a round clump of cells. The cell clumps, i.e., C-MSCs, were maintained in XF-CIM. C-MSCs generated with XF-CIM showed enlarged round cells, cartilage matrix, and hypertrophic chondrocytes genes elevation in vitro. Transplantation of C-MSCs generated with XF-CIM induced successful bone regeneration in the SCID mouse calvaria defect model. Immunofluorescence staining for human-specific vimentin demonstrated that donor human and host mouse cells cooperatively contributed the bone formation. Besides, the replacement of the cartilage matrix into bone was observed in the early period. These findings suggested that cartilaginous C-MSCs generated with XF-CIM can induce bone regeneration via endochondral ossification.

## 1. Introduction

Bone plays a significant role in supporting the body structure, shielding the vital organs, and providing minerals and blood cells to maintain homeostasis [1,2]. Although bones are comparatively regenerative tissues due to their unique remodeling system, large bone defects, pathological fractures, or inflammatory tissue destructive diseases such as rheumatoid arthritis and periodontitis induce irreversible lesions. Accordingly, the development of successful bone regenerative therapy for irreversible bone defects is great on demand.

One of the promising strategies for bone regenerative therapy includes the usage of mesenchymal stem cells (MSCs). MSCs, a class of adult stem cells, have attracted much medical and scientific attention for tissue regenerative therapy because of their self-renewing properties, multipotency, and trophic effects [3,4,5,6]. Especially, bone marrow-derived MSCs, which are relatively easily isolated, are well-utilized stem cells for bone regeneration, both in experiments and clinical practice [7,8,9]. Indeed, several tissue engineering approaches applying the MSCs by using the artificial scaffold for the bony defect have shown promising clinical results [10]. Nonetheless, several cases have, unfortunately, reported the opposite findings [11]. These inconsistent results may be attributed to some complications of artificial scaffold such as biodegradability and host unfavorable inflammation. Besides, the process of combining biomaterials and MSCs degrades the cell-cell or cell-extracellular matrix (ECM) contact, which may result in the disruption of the appropriate exercise of cellular function. To overcome these problems, great scientific efforts have been made to develop novel biomaterials that can mimic cellular microenvironment and bring out both grafted and host cells’ function [12,13,14]. These promising biomaterials will be applied to the clinical settings in the near future.

Otherwise, there is another strategy to avoid the obstacles regarding the biodegradability and unfavorable host metabolism of artificial scaffold; scaffold-free cell transplantation therapy. For the scaffold-free bone regenerative cell therapy, we have recently developed three-dimensional clumps of MSCs/ECM complexes (C-MSCs), which consisted of cells and self-produced ECM [15]. C-MSCs can be grafted into bone defects without an artificial scaffold to induce bone regeneration. In addition, the transplantation of C-MSCs, cultured with osteo-inductive medium (OIM) in vitro, showed greater bone regeneration in a rat cranial and a beagle dog periodontal tissue defect model [15,16]. These findings implied that C-MSCs cellular function regulated in vitro could be exerted at the transplanted site. More importantly, by using a SCID mouse calvarial defect model, we have demonstrated that human C-MSCs pretreated with OIM directly differentiate into osteocytes and deposit bone matrix proteins such as COL1, OPN, and OCN, to induce new bone formation in the grafted defect area [17]. In other words, the bone regeneration caused by C-MSCs treated with OIM could be due to an intramembranous ossification.

Although the traditional approach for bone regenerative cell therapy using osteo-inductive factors and biomaterials has mainly depended on this intramembranous ossification (or direct osteogenesis), there is a fact that most bone formation in the developing process or fracture healing proceeds via endochondral ossification [18]. During endochondral bone development, MSCs condense and undergo chondrogenesis to develop a cartilaginous template of the future bone. When the progenitor cells in the cartilage differentiate into late (“terminally differentiated”) hypertrophic chondrocytes, characterized by their enlarged round shape and collagen type X (ColX) production, the cartilage matrix is degraded by osteoclasts invading together with osteoprogenitors and thereby replaced by bone [19,20]. Importantly, hypertrophic chondrocytes are resistant to the low oxygen and nutrient microenvironment and stimulate both vascular endothelial cells and osteoblasts activity. Accordingly, tissue engineering therapy, which mimics the endochondral ossification by hypertrophic chondrocytes and cartilage matrix, can be a better candidate for the irreversible severe bone defect. In fact, previous studies demonstrated that subcutaneous transplantation of MSCs constructs, directed into chondrogenesis in vitro, can induce ectopic bone-like tissue formation in vivo [21,22,23]. Moreover, implantation of the cartilaginous pellet derived from human MSCs connected the mice segmental tibial defect with unmature bone like tissue [24]. Very recently, mechanical loading accelerates endochondral ossification by transplanted mesenchymal condensation, and thereby induces tibial segmental bone regeneration in the tibia segmental defect mice model [25]. However, it is still to be elusive whether chondrogenic MSCs constructs can successfully induce the bone regeneration in the calvarial defect model, of which healing process is mainly due to not endochondral but intramembranous ossification.

Notably, our recent study unveiled that C-MSCs are preferentially directed into chondrogenesis instead of osteogenesis in vitro due to the reduced YAP/TAZ mechanotransduction activity caused by the 3D floating-culture microenvironment [26]. Based on these accumulating lines of evidence, we hypothesized that C-MSCs cultured with chondro-inductive medium (CIM) could be cartilage anlagen, of which transplantation into bone defect can exert endochondral ossification to induce successful bone regeneration. Thus, in this present study, considering the future clinical application, we generated C-MSCs by xeno-free/serum-free (XF) CIM and assessed their bone regenerative property using the SCID mice calvarial defect transplantation model.

## 2. Materials and Methods

### 2.1. Generation of Human C-MSCs and Culture

Human C-MSCs were generated with XF condition as previously reported [17]. Briefly, passage number 5 (population doubling number is approximately 7 to 8) of commercially available human bone marrow MSCs (26 years old male donor; LONZA, Basel, Switzerland) were seeded at a density of 1.0 × 10^5^ cells/well in 48-well plates (Corning, Corning, NY, USA) and maintained in Prime-XV^®^ MSC expansion XSFM (GM) (Irvine Scientific, Santa Ana, CA, USA) for 4 days. To obtain C-MSCs, confluent cells that had formed on the cellular sheet, consisting of the ECM produced by MSCs themselves, were scratched by using a micropipette tip and then torn off. The MSC/ECM complex was detached from the bottom of the plate in a sheet shape and transferred to a 24-well ultra-low-binding plate (Corning). Then, the cellular sheet rolled up to make a round clump of cells, so called C-MSCs. The cell clumps were cultured in GM or MSCgo™ Chondrogenic XF medium (CIM) (Biological Industries, Beit Haemek, Israel) for 3, 5, 7, 10, 13, and 15 days.

### 2.2. Histological Analysis of-MSCs

C-MSCs were fixed with 4% paraformaldehyde in PBS. The samples were embedded in paraffin and 8-μm-thick semi-serial sections were prepared. The samples were then stained with hematoxylin/eosin (HE) or safranin O/fast green, and observed using a light microscope.

### 2.3. Real-Time Polymerase Chain Reaction

Total RNA from each cultured C-MSCs was extracted using RNA-iso^®^ (Takara, Otsu, Japan) and quantified by spectrometry at 260 and 280 nm. First-strand complementary DNA was synthesized with 500 ng of total RNA using ReverTraAce (Toyobo, Osaka, Japan). Then, real-time PCR was performed in a StepOne™ system (Applied Biosystems, Waltham, MA, USA) using SYBR green (Roche Applied Science, Mannheim, Germany) to determine the relative mRNA expression of sex determining region Y-Box 9 (*SOX9*), aggrecan (*ACAN*), collagen type II alpha 1 chain (*COL2A1*), collagen type X alpha 1 chain (*COL10A1*), and Indian hedgehog (IHH). The amplification conditions were as follows: 95.0 °C for 10 min, followed by 40 cycles at 95.0 °C for 15 s, and 60.0 °C for 1 min. Fold changes of the gene of interest were calculated with ∆∆Ct method by using *18S* as a reference control. The sequences of the primers used in this study are listed in Table S1.

### 2.4. Surgical Procedures

Seventy-two male NOD/SCID mice (7–8 weeks old) (Charles River Laboratories Japan, Yokohama, Japan), which were the fewest number of animal possible, were employed as a cranial defect model after approval had been obtained from the Animal Care Committee of Hiroshima University (protocol number: A18–180). The animals were maintained in a vivarium, with the room temperature set at 22 ± 2 °C and a 12-h light/dark cycle (lights on/off at 8:00 a.m./8:00 p.m.), and were given ad libitum access to food and water. Surgery was performed under general anesthesia with an intraperitoneal injection of medetomidine (0.3 mg/kg), midazolam (4 m/kg), and butorphanol tartrate (5 mg/kg). The skin at the surgical site was shaved and disinfected, and a sagittal skin incision was made from the occipital to the frontal bone. The skin flap, including the periosteum, was then dissected and elevated. Avoiding the cranial suture, two calvarial defects of 1.6 mm diameter were created in the left/right parietal bones at 3 mm lateral and 3 mm posterior to the bregma as a reference. One C-MSCs cultured with GM or CIM for 5, 10, or 15 days was transplanted into the defect with no artificial scaffold, respectively. A no implant group was also included as a control. Considering each defect as a sample, the no implant group or the different experimental groups were set and monitored for 4, 8, and 12 weeks (*n* = 6/each group). Using eighteen defects from 9 animals, C-MSCs cultured with CIM for 10 days were grafted and assessed for 3, 7, and 14 days as the healing process analysis. The skin was then closed using 4-0 silk suture. This animal study was performed in accordance with ARRIVE (Animal Research: Reporting In Vivo Experiments) guidelines.

### 2.5. Micro-CT Analysis

Mice were sacrificed at 4, 8, and 12 weeks after surgery, and the cranial region was imaged by using a SkyScan1176 in vivo μCT (Bruker, Billerica, MA, USA) with the following conditions: 50 kV, 0.5 mA, 8 µm pixel size, and 0.5 degree rotation step with 230 ms exposure time. Three-dimensional reconstructions were generated using CTVOL software (Bruker, Billerica, MA, USA). The region of interest (ROI) for bone volume measurement was the 1.6-mm circle of the bone defect that consists of 25 2D slices (approximately 450 µm thickness). Segmentation of the ROI and following bone volume measurement were performed by CT-An software (Bruker, Billerica, MA, USA) with a threshold range of 80–255 [17].

### 2.6. Tissue Preparation and Histological Analysis

The animals were sacrificed at 3 days, 1, 2, 4, 8, and 12 weeks after surgery. Cranial bones were harvested, fixed with 4% paraformaldehyde overnight, and decalcified with 10% ethylenediaminetetraacetic acid (pH 7.4) for 7 days. After decalcification, the samples were embedded in Tissue-Tek OCT compound (Sakura, Torrance, CA, USA). Semi-serial sections (8 μm) were cut in the frontal plane using a cryostat. These sections, showing the central portion of the bone defect, were stained with HE, safranin-O/fast green, or azocarmine G/aniline blue (AZAN), and observed using a light microscopy. To detect the human vimentin expression in the tissue, immunofluorescence analysis was performed. Briefly, the serial sections (20 μm) were incubated in LAB solution (Polyscience, Warrington, PA, USA) for 15 min at room temperature to activate antigens and were blocked with 5% BSA/0.1% Triton X-100/PBS blocking solution at room temperature for 1 h. These sections were then incubated with a rabbit anti-human vimentin IgG antibody (clone SP20, 1:100, Abcam, Cambridge, MA, USA) at 4 °C overnight. After being washed 3 times with PBS, samples were treated for 2 h with an Alexa Fluor 488_®_ goat anti-rabbit IgG antibody (1:100, Invitrogen, Carlsbad, CA, USA). Nuclei were counter-stained with DAPI (5 μg/mL; Invitrogen). After washing the samples with PBS, fluorescence signals were detected using the Olympus FV1000D laser scanning confocal microscope (Olympus, Tokyo, Japan).

### 2.7. Statistical Analysis

Concerning in vitro data, each group is represented by experimental replicates of a single MSCs preparation. In vivo data showed groups with different donors. Experiments were repeated three times, and the results are expressed as the means ± SD. Shapiro–Wilk test for distribution normality was conducted for each data set. Statistical analysis was performed using a two-tailed unpaired Student’s *t*-test to compare two different groups. To compare more than three different groups, one-way ANOVA with Tukey–Kramer post-hoc was conducted. Values of *p* < 0.05 or *p* < 0.01 were considered significant.

## 3. Results

### 3.1. Generation of Cartilaginous Tissue including Hypertrophic Chondrocyte from C-MSCs by Using XF-CIM

**Figure 1:**
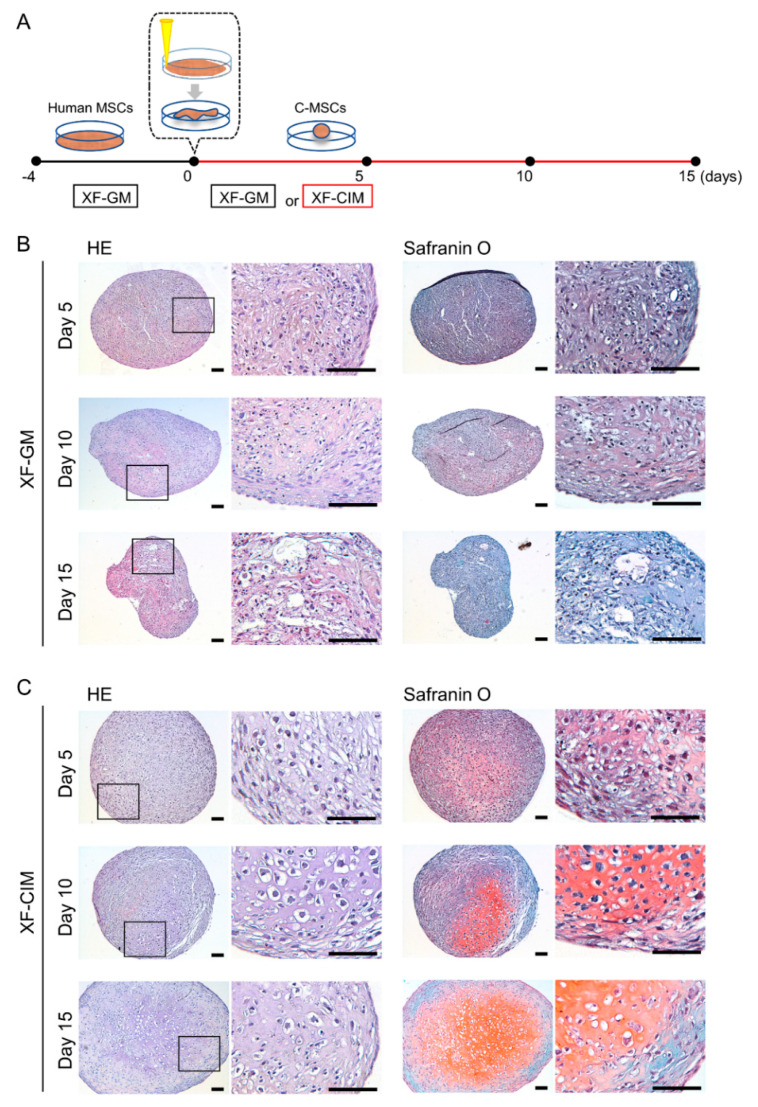
C-MSCs generated with xeno-free chondro-inductive medium show cartilaginous tissue. (**A**) Schematic figure of C-MSCs generation. (Figure 1B,C) C-MSCs were generated and maintained in XF-GM (**B**) or XF- CIM (**C**) for 5, 10, or 15 days as indicated. Semi-serial sections (8 μm) were stained with HE or safranin O/fast green, respectively. The left panels show lower magnification and magnified images in the boxed regions are indicated in the right panels. Bar = 100 μm.

Human C-MSCs were prepared from MSCs/ECM cellular sheet as described in the Materials and Methods section (Figure 1A). Consistent with our previous report, C-MSCs cultured with GM, mainly composed of fibrous ECM and cells, shrank in a time dependent manner [15]. The cell clumps were not stained with safranin O during the culture period (Figure 1B). Otherwise, C-MSCs cultured with CIM for 5 days showed slight stained safranin O and a few round cells. Moreover, maturated cartilaginous ECM, as indicated by intense staining with safranin O, was observed in the center of C-MSCs on days 10 and 15. Interestingly, there were enlarged round cells in the cartilage matrices (Figure 1C).

**Figure 2:**
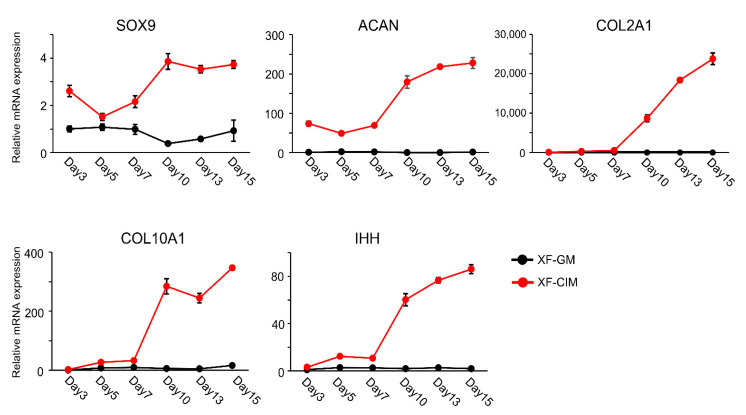
C-MSCs generated with XF-CIM expressed hypertrophic chondrocytes marker genes.

Then, we investigated the chondrogenic marker genes expression pattern in C-MSCs during CIM culture (Figure 2). Compared to C-MSCs cultured with GM, chondrocyte markers, SOX9, ACAN, and COLII mRNA expression levels were higher in C-MSCs cultured with CIM, and were increasing in a time dependent manner. Besides, as the expression levels of COLX and IHH were also up-regulated, chondrocyte hypertrophy appeared to be induced in C-MSCs cultured with CIM. More specifically, all tested chondrocyte marker genes were drastically elevated from day 7 to day 10, reflecting the histological analysis of C-MSCs for safranin O staining in Figure 1. Taken together, these findings in Figure 1 and Figure 2 indicated that CIM induced C-MSCs into chondrogenesis and hypertrophic differentiation occurred around day 10.

C-MSCs were cultured in XF-GM or XF-CIM for the indicated culture period. The expression levels of chondrocyte marker genes were analyzed by real-time PCR with ∆∆Ct method by using *18S* as a reference control. Data were normalized to the values of C-MSCs maintained in XF-GM for 3 days as 1.0. Values represent means ± S.D. of three replicates of culture. The black line indicates XF-GM-treated group, and the red line is XF-CIM cultured one. All graphs are representative of four independent experiments.

### 3.2. Transplantation of C-MSCs Cultured with XF-CIM Exerts Effective Bone Regenerative Property

**Figure 3:**
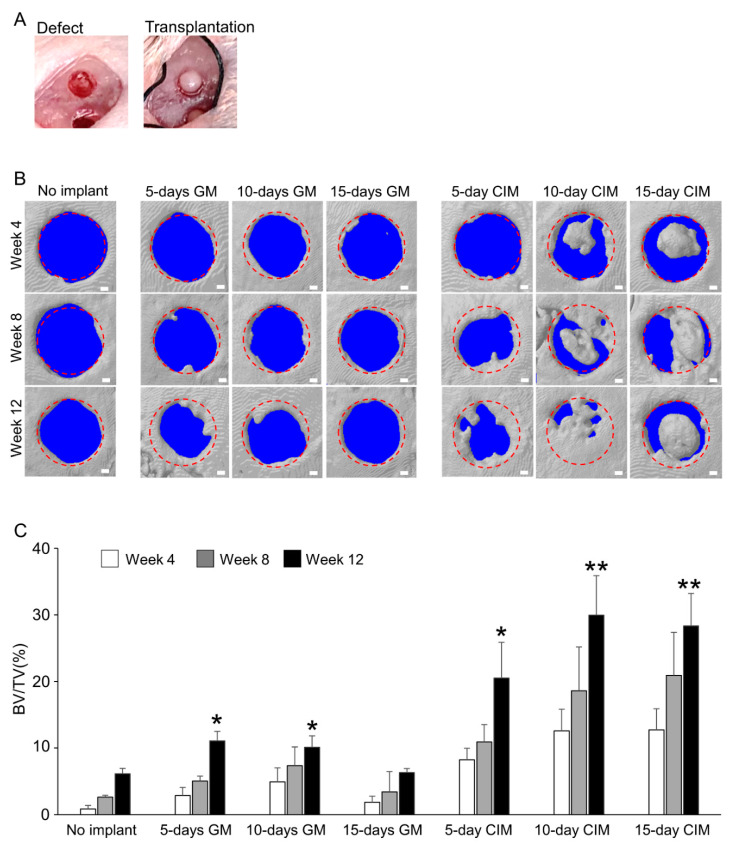
C-MSCs generated with XF-CIM expressed hypertrophic chondrocytes marker genes. (**A**) C-MSCs cultured in XF-GM or XF-CIM for 5, 10, or 15 days were directly transplanted into a SCID mouse cranial defect 1.6 mm in diameter. No implant group was set as a control (*n* = 6/each group). (**B**) Representative micro-CT images of six samples at 4, 8, and 12 weeks surgery. Bar = 250 μm. (**C**) Ratio of the segmented bone volume (BV) to the total volume (TV) of the defect region after 4, 8, and 12 weeks of surgery. Values are mean ± S.D. of six mice per group, * *p* < 0.05, ** *p* < 0.01, and differ significantly from the no implant group (ANOVA). 5-day GM: transplantation of C-MSCs cultured in XF-GM for 5 days; 10-day GM: transplantation of C-MSCs cultured in XF-GM for 10 days; 15-day GM: transplantation of C-MSCs cultured in XF-GM for 15 days; 5-day CIM: transplantation of C-MSCs cultured in XF-CIM for 5 days; 10-day CIM: transplantation of C-MSCs cultured in XF-CIM for 10 days; 15-day CIM: transplantation of C-MSCs cultured in XF-CIM for 15 days.

To investigate whether chondro-induction in vitro can increase the bone-regenerative properties of C-MSCs, C-MSCs generated with each culture condition (XF-GM for 5, 10, 15 days, XF-CIM for 5, 10, and 15 days, respectively: *n* = 6/each group) were directly grafted into SCID mice cranial defects with no artificial scaffold (Figure 3A). Micro-CT 3D-reconstructed images showed unsuccessful bone regeneration during the experimental periods in no implant group. C-MSCs cultured in GM for 5 or 10 days and CIM for 5 days slightly induced bone regeneration from the edge of the defects after 12 weeks of transplantation, respectively (Figure 3B,C). Transplantation of C-MSCs treated with GM for 15 days failed to induce bone regeneration (Figure 3B,C). On the other hand, implantation of C-MSCs generated with CIM for 10 or 15 days led to the bone formation from the inside of the defect on week 4 (Figure 3B upper panels) and successfully induced bone regeneration on week 12 (Figure 3B,C). These findings suggested that cartilage-like C-MSCs possesses greater bone regenerative capacity than that of un-differentiated C-MSCs.

### 3.3. Cartilage-like C-MSCs Generated with CIM but Not Unmatured Ones induce Donor and Host Cells Cooperative Bone Formation

**Figure 4:**
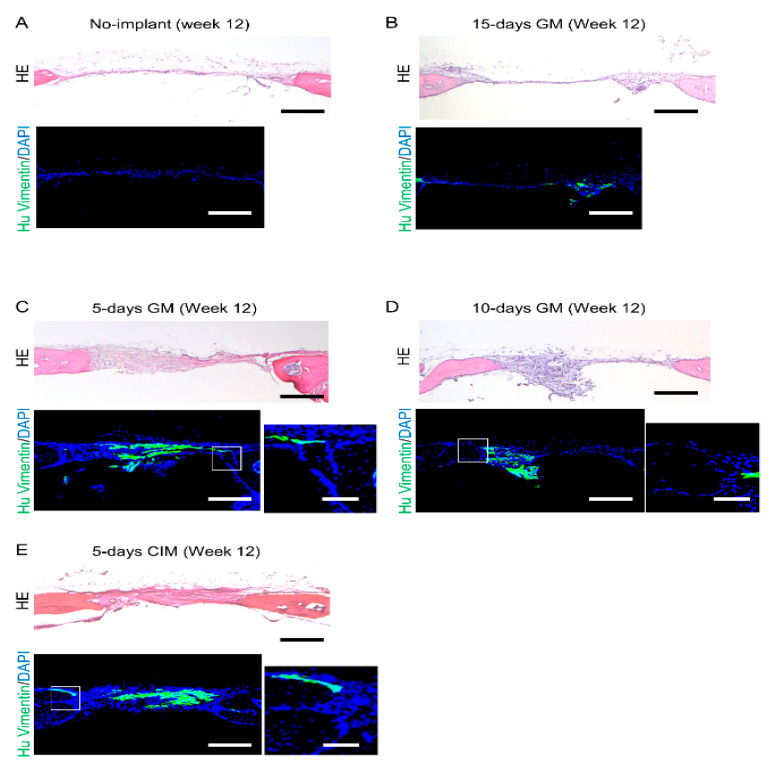
Transplantation of C-MSCs cultured with XF-GM for 5 or 10 days, or XF-CIM for 5 days slightly induces host new bone formation. (**A**–**E**) Animals were sacrificed at 12 weeks after surgery and the cranial bones were isolated. Semi-serial sections (8 μm) were obtained and stained with HE and immunostained with anti-human vimentin antibody, as indicated. Nuclei were counterstained with DAPI for immunostaining. HE and left panels of immunostaining images show lower magnification, Bar = 250 μm. Right panels of immunostaining indicate higher magnification, Bar = 50 μm. (**A**) No implant. (**B**) 15-days GM: transplantation of C-MSCs cultured in XF-GM for 15 days. (**C**) 5-days GM: transplantation of C-MSCs cultured in XF-GM for 5 days. (**D**) 10-days GM: transplantation of C-MSCs cultured in XF-GM for 10 days. (**E**) 5-days CIM: transplantation of C-MSCs cultured in XF-CIM for 5 days. All images are representative of six samples.

To assess the role of donor and host cells in the new bone formation caused by C-MSCs transplantation, histological analyses were conducted. Consistent with the micro CT data, at 4 and 8 weeks after transplantation of C-MSCs generated with XF-GM for 5, 10, 15 days or XF-CIM for 5 days, new bone formation was not observed at the defect area (Figures S1 and S2). After 12 weeks of surgery, HE staining demonstrated only thin, soft fibrous tissue connecting the defect edges in the no graft group and C-MSCs cultured with XF-GM for 15 days transplantation group (Figure 4A,B). On the other hand, C-MSCs cultured with XF-GM for 5 or 10 days, or XF-CIM for 5 days induced slight new bone formation from the periphery of the defects after 12 weeks of transplantation. However, most of the defects was filled with thick connective tissue (Figure 4C–E). Immunofluorescence staining using human vimentin specific antibody showed that human donor cells were mainly observed in the connective fibrous tissue, and vimentin negative host mouse cells composed of the newly formed bone of the defect edge (Figure 4C–E). A few vimentin-positive donor cells were lining on the surface of new bone from the periphery of the defect (higher magnification images of Figure 4C–E).

**Figure 5:**
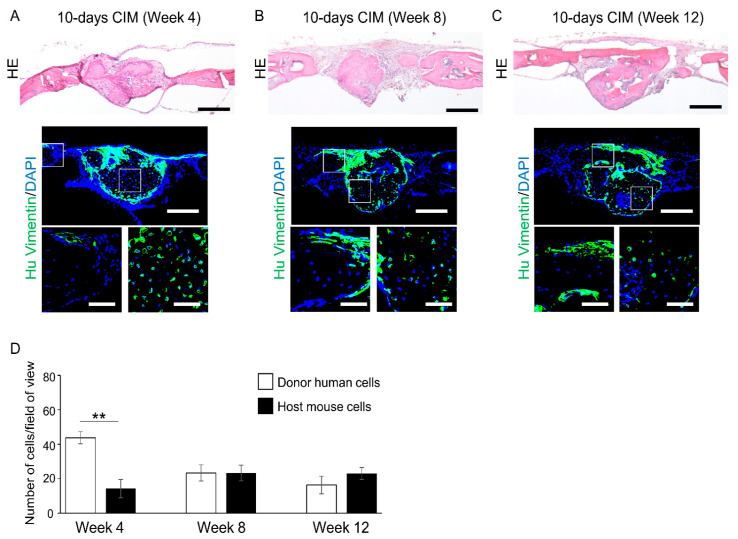
Transplantation of cartilage-like C-MSCs cultured with XF-CIM for 10 days facilitates donor and host cells cooperative bone formation. (**A**–**A**) C-MSCs cultured with XF-CIM for 10 days were directly transplanted into a SCID mouse cranial defect 1.6 mm in diameter. Animals were sacrificed at 4 (**A**), 8 (**B**), and 12 weeks (**C**) after surgery and the cranial bones were fixed. Semi-serial sections (8 μm) were stained with HE and immunostained with anti-human vimentin antibody, as indicated. Nuclei were counterstained with DAPI for immunostaining. HE and upper panels of immunostaining images show lower magnification. Bar = 250 μm. Magnified immunostained images in the boxed regions are indicated at the bottom panels. Bar = 50 μm. 10-days CIM: transplantation of C-MSCs cultured in XF-CIM for 10 days. All images are representative of six samples. (**D**) Four higher magnification views in the new bone observed at the defect center (descended from C-MSCs) were used for counting of human vimentin-positive (donor human) and -negative (host mouse) cells. Values are expressed as means ± S.D. of the four views tested for each group. ** *p* < 0.01.

Four weeks after transplantation of C-MSCs cultured with XF-CIM for 10 days, round shaped bone-like tissue well stained with eosin was observed at the center of the defect (Figure 5A). Besides, this bone-like tissue was connected with thin new bone extending from the peripheries of the defect after 12 weeks of transplantation (Figure 5C). At 4 weeks of transplantation, the newly formed bone at the center of the defect mainly consisted of human vimentin-positive cells, suggesting grafted human C-MSCs (Figure 5A,D). Then, the number of human donor cells decreased in a time-dependent manner, and human vimentin negative host mouse cells were mainly observed in the new bone filling the defect at 12 weeks after implantation (Figure 5A–D). On the other hand, new bone extended from the defect edge was covered with human vimentin-positive donor cells in a time-dependent manner (Figure 5A–C). Similar results were observed when C-MSCs cultured with CIM for 15 days were grafted (Figure S3). These findings suggested that transplantation of cartilaginous C-MSCs induces bone regeneration by donor and host cells cooperative bone formation.

### 3.4. Transplantation of Cartilage-Like C-MSCs Generated with CIM Causes Endochondral Ossification to Facilitate Bone Regeneration

**Figure 6:**
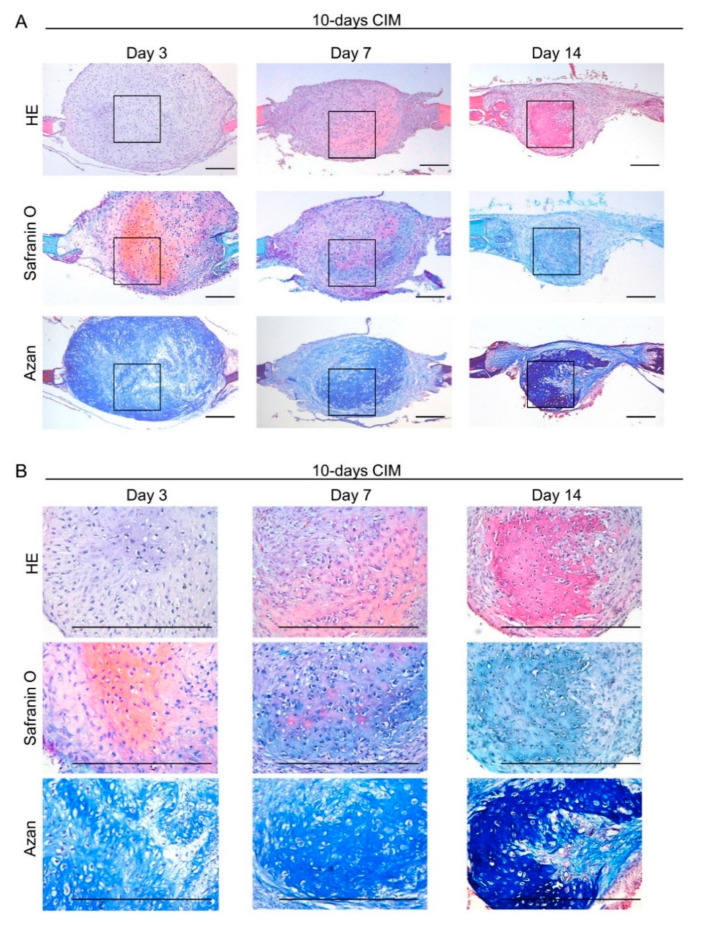
Transplantation of cartilage-like C-MSCs cultured with XF-CIM for 10 days causes endochondral ossification in the early period. (**A**,**B**) C-MSCs cultured with XF-CIM for 10 days were directly transplanted into a SCID mouse cranial defect of 1.6 mm in diameter. Eighteen defects were created in 9 animals. Animals were sacrificed at 3, 7, and 14 days after surgery and the cranial bones were fixed (*n* = 6/each experimental periods). Semi-serial sections (8 μm) were stained with HE, safranin O/fast green and azan, as indicated. (**A**) Lower magnification (Bar = 250 μm). (**B**) Magnified images in the boxed regions (Bar = 50 μm). 10-days CIM: transplantation of C-MSCs cultured in XF-CIM for 10 days. All images are representative of six samples.

**Figure 7:**
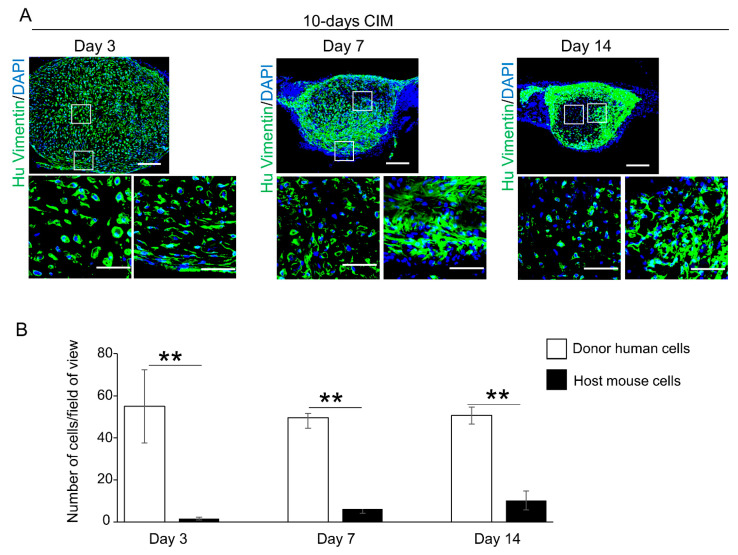
Transplanted donor cells may be associated with the new bone-like tissue formation in the process of endochondral ossification by cartilage-like C-MSCs transplantation. (**A**). Semi-serial sections (8 μm) obtained from Figure 6 experiments were immunostained with anti-human vimentin antibody. Nuclei were counterstained with DAPI. The upper panels indicate lower magnification (Bar = 250) and higher magnified images in the boxed regions are indicated at the bottom panels, respectively. All images are representative of six samples. Bar = 50 μm. 10-days CIM: transplantation of C-MSCs cultured in XF-CIM for 10 days. (**B**) Four higher magnification views at the center of transplanted C-MSCs (the area replacing cartilage to the bone-like matrix) were used for counting of human vimentin-positive (donor human) and -negative (host mouse) cells. Values are express as means ± S.D. of the four views tested for each group. ** *p* < 0.01.

At week 4, cartilaginous C-MSCs (generated from 10 or 15 days of XF-CIM culture), but not fibrous unmatured ones (generated from 5 days of XF-GM or XF-CIM culture), induced bone-like tissue in the defect center (Figure 3). More specifically, since the new bone mainly consisted of human donor cells, it could have descended from implanted C-MSCs (Figure 4A). Besides, the hypertrophic cartilage-like structure was included inside of the C-MSCs generated with CIM for 10 or 15 days (Figure 1). Based on these findings, we speculated that the new bone formation at the center of the defect was due to the endochondral ossification from the transplanted cartilaginous C-MSCs. To test this hypothesis, in the following experiments, we focused on the tissue-healing process 3, 7, and 14 days after transplantation of C-MSCs cultured in XF-CIM for 10 days. After 3 days of C-MSCs transplantation, a cartilage matrix stained with safranin O was observed at the defect center (Figure 6A,B; middle panels). The safranin O-stained matrix reduced on day 7 and disappeared on day 14 (Figure 6A,B; middle panels). Moreover, HE staining indicated that the bone-like tissue formation occurred at the site where the cartilage matrix was degraded (Figure 6A,B; upper panels). This subsequent bone-like tissue generation in accordance with the cartilage degradation was also supported by the results of AZAN staining (Figure 6A,B; lower panels). Immunofluorescence analysis demonstrated that vimentin-negative mouse cells covered the grafted human C-MSCs and some host cells penetrated the human cell clumps on day 7 (Figure 7A; left and middle panels). Fourteen days after cartilaginous C-MSCs transplantation, the newly formed bone-like tissue as shown in Figure 6 was surrounded by the spindle-shaped human fibroblastic cells (Figure 7A; right panel). Interestingly, although there were host mouse cells, the majority of the new bone-like tissue was human donor derived (Figure 7B). These findings suggested that transplanted cartilaginous C-MSCs undergo endochondral ossification to induce bone regeneration. Besides, a part of grafted hypertrophic chondrocytes may transdifferentiate into osteogenic cells.

## 4. Discussion

Previous studies, which aimed to develop bone regenerative MSCs transplantation therapy, frequently employed pretreatment of osteo-induction. Such a strategy successfully induced bone regeneration via MSCs intramembranous ossification and paracrine effect [17,27,28]. Otherwise, in this present study, we applied chondrogenic induction to increase C-MSCs bone regeneration property. As a result, cartilaginous C-MSCs (generated with CIM for more than 10 days) facilitated bone regeneration in SCID mouse cranial defect model, and a part of the new bone was derived via endochondral ossification (Figure 5). Notably, both unmatured C-MSCs (cultured with GM for 5 or 10 days) and C-MSCs generated with CIM for 5 days, which did not undergo hypertrophic differentiation in vitro, induced only host bone formation from the periphery of the defect, suggesting that the undifferentiated cells slightly induce tissue regeneration via paracrine effect but not self-tissue formation (Figure 4). In addition, transplantation of C-MSCs generated with GM for 15 days, which was obviously smaller than the other cell clumps, failed to induce new bone formation (Figure 4). Accordingly, cartilaginous ECM and hypertrophic cells in C-MSCs generated with CIM for 10 days are key to induce successful bone regeneration.

Interestingly, grafted donor human cells but not host cells were mainly observed in the new bone as a result of endochondral ossification, which occurred at the center of the defect (Figure 6 and Figure 7). Besides, although the number of donor human cells decreased in accordance with the bone maturation, several human cells remained in the bone matrix (Figure 5). These findings suggested one possibility that grafted human hypertrophic chondrocytes could be transdifferentiated into osteogenic cells. Over a decade ago, in the endochondral ossification, it was believed that hypertrophic chondrocytes play a role in mineral deposition in the cartilage matrix and then undergo cell apoptosis. Then, osteoblastic cells surrounding the hypertrophic cartilage took the place of dead hypertrophic chondrocytes, which, in turn, results in the new bone formation [18]. However, recent studies clearly demonstrated that a part of hypertrophic chondrocyte could survive and transdifferentiate into osteoblastic cells to form long bone in the endochondral ossification of the development process [29,30,31]. Besides, Bahney et al. reported that transplantation of cartilage, obtained from fracture callus of LacZ+/+ Rosa26 reporter mice, causes bone regeneration via endochondral ossification in a SCID mice segmental bone defect model. More importantly, the majority of the bone regeneration was from LacZ positive donor, rather than host derived, suggesting the chondrocytes transdifferentiation [24]. Taken together, supporting these recent reports, we speculate hypertrophic chondrocytes in cartilaginous C-MSCs transdifferentiate into osteogenic cells to induce bone regeneration via endochondral ossification. To clarify this speculation, future study detecting osteogenic markers expression in donor cells should be required.

In general, to investigate the MSCs chondrogenic differentiation, MSCs spheroid, which consisted of cells that organize themselves via cell–cell contact, is employed. On the other hand, C-MSCs are composed of cells and self-produced ECM. This discrepancy may affect the biological property of the cartilaginous product derived by chondrogenic induction. Indeed, cartilage pellet obtained from MSCs spheroid shows uniform and wide cartilage matrix distribution, whereas cartilaginous C-MSCs demonstrates not only cartilage matrix but also abundant fibrous tissue surrounding the cartilage. Although the cellular property of the fibroblasts in cartilaginous C-MSCs is unclear, those cells may retain the mesenchymal stromal/stem cells function that supports the bone formation/remodeling. The periosteum, consisting of connective tissue, contains skeletal stem cells that maintain bone homeostasis [32]. Accordingly, there is a possibility that the fibroblastic cells forming the outer of cartilaginous C-MSCs also might play a similar role in the bone regeneration like skeletal stem cells in periosteum, though additional study investigating its cellular function will be needed.

Previously, we have generated C-MSCs by using XF-OIM. After 8 weeks of transplantation of OIM-treated C-MSCs, complete bone regeneration was observed in the SCID mouse cranial defect model, and the new bone is attributed to the donor and host cells intramembranous ossification [17]. Briefly, C-MSCs generated with OIM seems to be superior to CIM-generated C-MSCs regarding the bone regenerative pace. However, it is reported that chondrocytes are resistant to a hypo-nutrition situation because physiological chondrocytes exist in a hypoxic environment within the avascular cartilage tissue [33]. This biological character could be advantageous for the larger bone defect case, of which defect center is hypo-nutrition condition. Indeed, in this present study, cartilaginous C-MSCs can induce new bone formation far from the defect edge via endochondral ossification by themselves. Similar endochondral ossification by cartilaginous C-MSCs could be expected in the larger defect model. Accordingly, to apply OIM-treated or CIM-treated C-MSCs as the situation demands may be important. Alternatively, a combined usage could also be a good strategy for a large defect.

Although this present study grafted one C-MSCs into a small bone defect, we have previously demonstrated that multiple number of C-MSCs can be piled up in large and complicated tissue defects. For instance, 48 C-MSCs generated with OIM were grafted into beagle dog class III furcation defect to induce successful bone regeneration [16]. However, for C-MSCs transplantation therapy, larger bone defect cases in clinical settings could still be a challenge. To treat a segmental tibial fracture with a 4-cm gap, approximately 200–300 C-MSCs will be required. It should be hard for the grafted C-MSCs to remain in such a large defect area to exert their bone regenerative property. To solve this potential problem, recent advanced technology of the Bio 3D computer-controlled printing could be applicable [34]. Large cell constructs can be produced by a bio-3D printer using cell aggregates composed of various cells that can contribute to tissue reconstruction, including articular cartilage [35,36]. Indeed, Mituzawa et al. have developed an artificial scaffold-free 3D nerve conduit by using the bio-3D printer and cell clumps generated with iPS-derived MSCs, so called C-iMSCs. The 3D conduit was composed of approximately 500 C-iMSCs, and its transplantation induced peripheral nerve regeneration [37]. Thus, using the bio-3D printer, we may generate a cm order of cartilaginous C-MSCs, which can be applicable to the larger bone defect cases in clinical orthopedics.

Fetal bovine serum (FBS) is frequently used for biological studies because it contains various beneficial biomolecules for cell cultures. However, its clinical application raises several concerns, such as microbiological contamination, potential transmission of animal disease, and high variability between batches [38,39]. To avoid this problem regarding FBS usage in the clinical situation, in this present study, we have generated C-MSCs with XF condition. Besides, we have previously demonstrated that C-MSCs can be cryopreserved with XF condition [40]. Importantly, it is reported that both MSCs and chondrocytes show low immunogenicity and high immunomodulatory property to avoid allograft immune rejection [41,42,43,44]. These facts imply that cartilaginous C-MSCs allograft may be applicable for clinical bone regenerative therapy.

## 5. Conclusions

In conclusion, we generated cartilaginous C-MSCs by using XF-CIM. Its transplantation with no artificial scaffold induced successful bone regeneration in a SCID mouse cranial defect model. Besides, a part of the new bone formation is due to endochondral ossification. Since previous studies have mainly employed osteo-induction, our findings focusing on chondro-induction may shed light on the novel strategy to develop promising bone regenerative therapy by using MSCs.

## Data Availability

The datasets used or analyzed during the current study available from the corresponding author on reasonable request.

## Supplementary Materials

The following are available online at https://www.mdpi.com/article/10.3390/biomedicines9101408/s1. Figure S1: Histological evaluation at 4 weeks after unmature C-MSCs transplantation. Figure S2: Histological evaluation at 8 weeks after unmature C-MSCs transplantation. Figure S3: Transplantation of C-MSCs cultured with XF-CIM for 15 days activates donor and host cells corporative bone formation. Table S1: Sense and antisense primers for real-time PCR.
