# Supplementary material for: Clumps of Mesenchymal Stem Cells/Extracellular Matrix Complexes Generated with Xeno-Free Chondro-Inductive Medium Induce Bone Regeneration via Endochondral Ossification
Source: Biomedicines. 2021 Oct 7;9(10):1408. doi: 10.3390/biomedicines9101408 (PMC8533314; doi:10.3390/biomedicines9101408)
Supplement: Supplementary file 1 [file biomedicines-09-01408-s001.zip › supplementary figure S1.pdf]

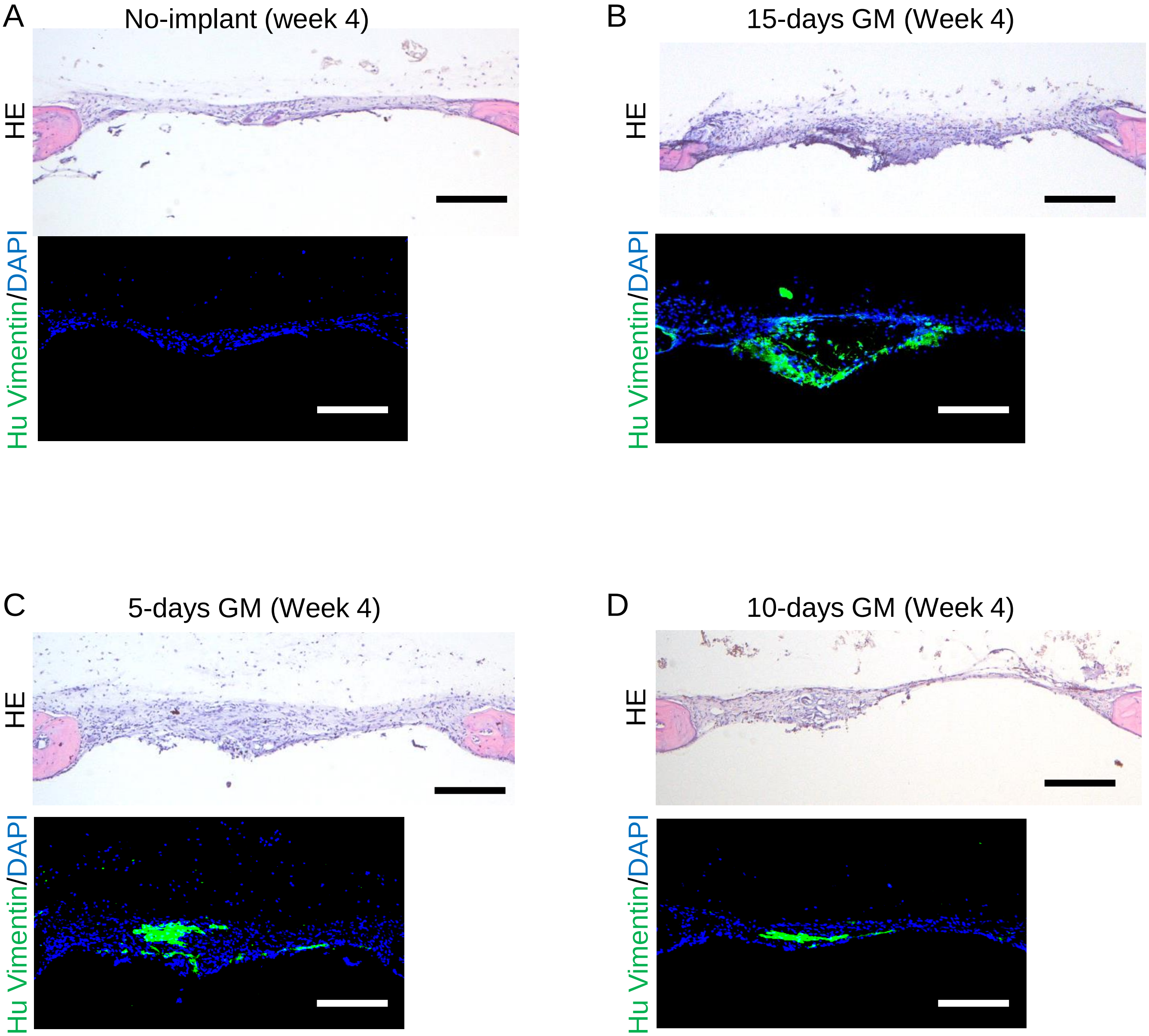

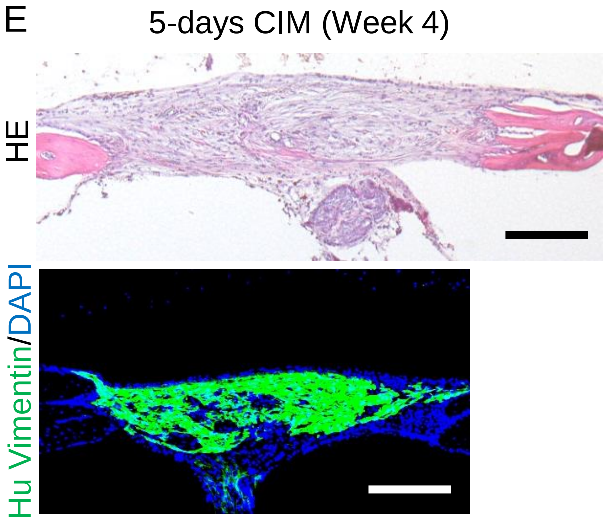

Supplementary Figure S1. Histological evaluation at 4 weeks after unmature C-MSCs transplantation.

(A-E) Animals were sacrificed at 4 weeks after surgery and the cranial bones were isolated. Semi-serial sections (8 μm) were obtained and stained with HE and immunostained with anti-human vimentin antibody, as indicated. Nuclei were counterstained with DAPI for immunostaining. Bar = 250 μm. (A) No implant. (B) 15-days GM: transplantation of C-MSCs cultured in XF-GM for 15 days. (C) 5-days GM: transplantation of C-MSCs cultured in XF-GM for 5 days. (D) 10-days GM: transplantation of C-MSCs cultured in XF-GM for 10 days. (E) 5-days CIM: transplantation of C-MSCs cultured in XF-CIM for 5 days. All images are representative of six samples.
