# Supplementary material for: Clumps of Mesenchymal Stem Cells/Extracellular Matrix Complexes Generated with Xeno-Free Chondro-Inductive Medium Induce Bone Regeneration via Endochondral Ossification
Source: Biomedicines. 2021 Oct 7;9(10):1408. doi: 10.3390/biomedicines9101408 (PMC8533314; doi:10.3390/biomedicines9101408)
Supplement: Supplementary file 1 [file biomedicines-09-01408-s001.zip › supplementary figure S3.pdf]

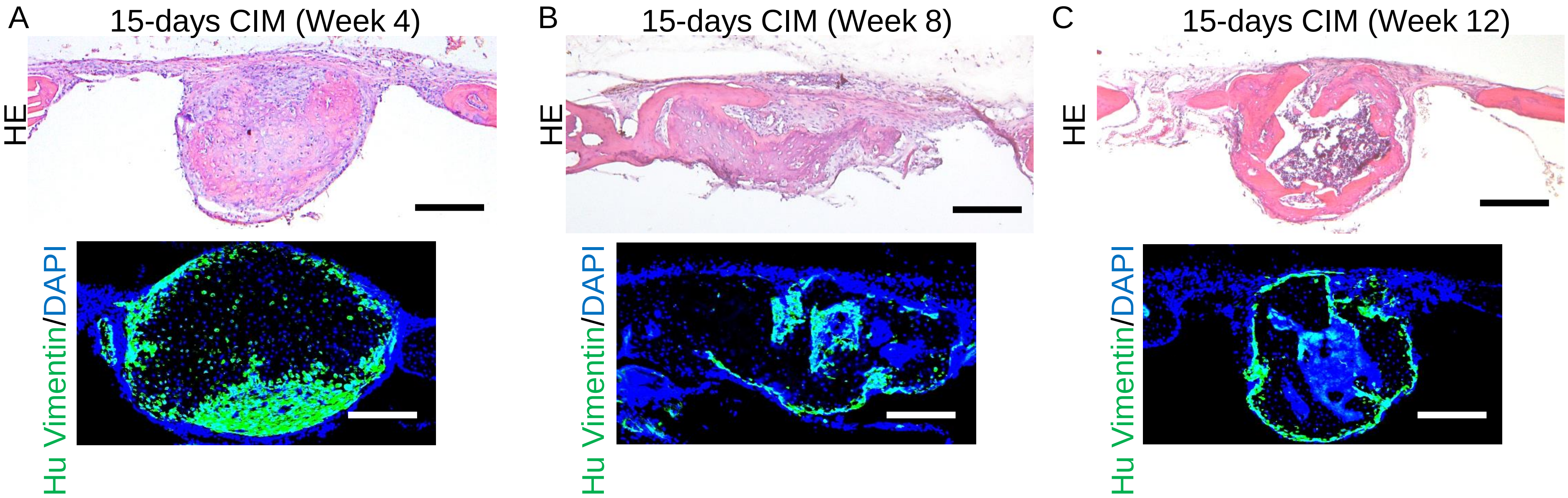


**Supplementary Figure S3.** Transplantation of C-MSCs cultured with XF-CIM for 15 days activates donor and host cells cooperative bone formation.(A-C) C-MSCs cultured with XF-CIM for 10 days were directly transplanted into a SCID mouse cranial defect 1.6 mm in diameter. Animals were sacrificed at 4 (A), 8 (B), and 12 weeks (C) after surgery and the cranial bones were fixed. Semi-serial sections were stained with HE and immunostained with anti-human vimentin antibody, as indicated. Nuclei were counterstained with DAPI for immunostaining.
