# Supplementary material for: Clumps of Mesenchymal Stem Cells/Extracellular Matrix Complexes Generated with Xeno-Free Chondro-Inductive Medium Induce Bone Regeneration via Endochondral Ossification
Source: Biomedicines. 2021 Oct 7;9(10):1408. doi: 10.3390/biomedicines9101408 (PMC8533314; doi:10.3390/biomedicines9101408)
Supplement: Supplementary file 1 [file biomedicines-09-01408-s001.zip › supplementary table S1.pdf]

Supplementary Table S1. Sense and antisense primers for real-time PCR

Target gene		*Primer sequence*
Sox9	Forward	5'- CATGAGCGAGGTGCACTCC -3'
	Reverse	5'- TCGCTTCAGGTCAGCCTTG -3'
ACAN	Forward	5'- TGAGGAGGGCTGGAACAAGTACC -3'
	Reverse	5'- GGAGGTGGTAATTGCAGGGAACA -3'
COL2A1	Forward	5'- TTTCCCAGGTCAAGATGGTC -3'
	Reverse	5'- CTTCAGCACCTGTCTCACCA -3'
COL10A1	Forward	5'- CCCTTTTTGCTGCTAGTATCC -3'
	Reverse	5'- CTGTTGTCCAGGTTTTCCTGGCAC -3'
IHH	Forward	5'- AACTCGCTGGCTATCTCGGT -3'
	Reverse	5'- GCCCTCATAATGCAGGGACT -3'
18S	Forward	5'- GTAACCCGTTGAACCCCATT -3'
	Reverse	5'- CCATCCAATCGGTAGTAGCG -3'
